# Increased incidence of *Mycoplasma pneumoniae* infections and hospital admissions in the Netherlands, November to December 2023

**DOI:** 10.2807/1560-7917.ES.2024.29.4.2300724

**Published:** 2024-01-25

**Authors:** Dita C Bolluyt, Sjoerd M Euser, Dennis Souverein, Annemarie MC van Rossum, Jayant Kalpoe, Mireille van Westreenen, Marco Goeijenbier, Dominic Snijders, Dirk Eggink, Femke Jongenotter, Steven FL van Lelyveld, Marlies A van Houten

**Affiliations:** 1 Department of Internal Medicine, Spaarne Gasthuis, Haarlem/Hoofddorp, the Netherlands; 2 Regional Public Health Laboratory Kennemerland, Haarlem, The Netherlands; 3 Department of Paediatrics, Erasmus MC University Medical Centre, Rotterdam, the Netherlands; 4 Department of Medical Microbiology, Erasmus MC University Medical centre, Rotterdam, the Netherlands; 5 Department of Intensive Care Medicine, Spaarne Gasthuis, Haarlem/Hoofddorp, the Netherlands; 6 Department of Intensive Care Medicine, Erasmus MC University Medical Centre, Rotterdam, the Netherlands; 7 Department of Pulmonary Medicine, Spaarne Gasthuis, Haarlem/Hoofddorp, the Netherlands; 8 Centre for Infectious Disease Control, National Institute for Public Health and the Environment, Bilthoven, The Netherlands; 9 Department of Paediatrics, Spaarne Gasthuis, Haarlem/Hoofddorp, The Netherlands; * These authors contributed equally to this work and share authorship.

**Keywords:** Mycoplasma pneumoniae, upsurge, the Netherlands, Europe

## Abstract

*Mycoplasma pneumoniae* is an important cause of pneumonia and extra-pulmonary manifestations. We observed a rise in admissions due to *M. pneumoniae* infections starting October 2023 in a regional hospital in the Netherlands and an increased incidence in national surveillance data. The incidence in the Netherlands has not been that high since 2011. The patients had a lower median age compared with 2019 and 2020 (28 vs 40 years). *M. pneumoniae *should be considered in patients with respiratory symptoms, especially children.

*Mycoplasma pneumoniae* is a common cause of lower and upper respiratory tract infections, especially in children and young adults. Extra-pulmonary symptoms such as erythema exsudativum multiforme and encephalitis are also reported [1,2]. Epidemics caused by *M. pneumoniae* are known to occur worldwide every few years [2,3], with the last observed epidemic in some European countries in 2019/20 [4]. After several years of low detection rates, we noted in the last months of 2023, a sharp local increase in *M. pneumoniae* detections as well *as M. pneumoniae*-related admissions in our hospital (Spaarne Gasthuis) in the Netherlands. Here, we aimed to report the current upward trend in *M. pneumoniae* detections in more detail and compare it with national and European trends.

## Local incidence and hospital admissions

To gain more detailed insight into the current *M. pneumoniae* epidemiology, we used data from the Regional Public Health Laboratory Kennemerland (RPHLK) that performs diagnostic tests for Spaarne Gasthuis (a secondary centre with 560 beds and 54,000 annual visits to the emergency department divided over two locations). We collected the number of *M. pneumoniae* detections (from 2017 until 2023, 257 detections out of 19,989 tests) and hospital admissions, as well as demographics data of the patients (i.e. age, sex (male/female), indication of admission, duration of admission, admission to an intensive care unit (ICU), and co-detections). Hospital admission related to *M. pneumoniae* was defined as the detection of *M. pneumoniae* in a naso- and/or oropharyngeal (NP/OP) swab using PCR-based diagnostic tests (as described before [5]) up to 2 days before or during admission. Furthermore, we compared the current data against those from 2019 and 2020, the last period with increased detection of *M. pneumoniae* in our hospital.

**Figure 1:**
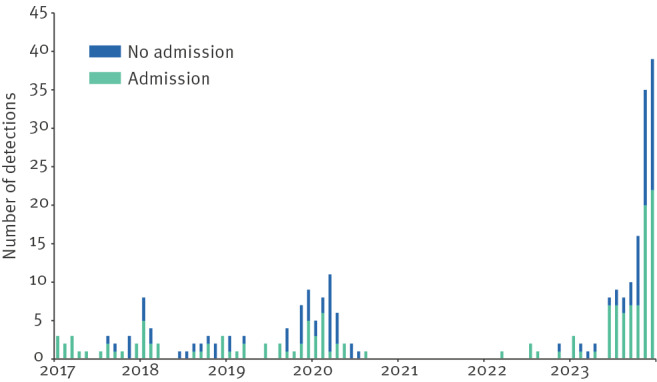
*Mycoplasma pneumoniae* detections in Spaarne Gasthuis hospital, Hoofddorp/Haarlem, the Netherlands, 2017–2023 (n = 257)

We observed a steep rise in regional *M. pneumoniae* detections in 2023, starting in October (Figure 1). In 2023, 133 patients with *M. pneumoniae*-positive PCR were detected in Spaarne Gasthuis, with a median age of 28 years (interquartile range (IQR): 9–43) (Table). Fifty-five (41.3%) patients were younger than 18 years, with most children in the age category 5–11 years (n = 27). In adults, most detections were found in the youngest age categories: 18–29 (n = 16) and 30-39 years (n = 22). Demographic data and case numbers in children vs adults are appended in Supplementary Table S1 and Figure S1. Comparing *M. pneumoniae* PCR-positivity between 2023 (4.1%; 133/3,227) and 2019 and 2020 (1.0%; 68/6,889) showed a significant increase (p < 0.001).

Detections of *M. pneumoniae* by PCR on naso- and oropharyngeal swab [5].

**Table:** Demographic data of patients with *Mycoplasma pneumoniae* detections in Spaarne Gasthuis hospital, Hoofddorp/Haarlem, the Netherlands 2019 and 2020 vs 2023 (n = 201)

	2019 and 2020 (n = 68)		2023 (n = 133)		Total (n = 201)		p value
	n	%	n	%	n	%	
**Sex (male/female)**							
Male	32	47.1	65	48.9	97	48.3	0.925
Female	36	52.9	68	51.1	104	51.7	
**Age (years)**							
Median (IQR)	40.0 (21.2–7.0)		28.0 (9.0–3.0)		33.0 (10.0–8.0)		0.005
**Age group (years)**							
0–4	5	7.4	14	10.5	19	9.5	0.129
5–11	8	11.8	27	20.3	35	17.4	
12–17	2	2.9	14	10.5	16	8.0	
18–29	7	10.3	16	12.0	23	11.4	
30–39	11	16.2	22	16.5	33	16.4	
40–49	15	22.1	15	11.3	30	14.9	
50–64	6	8.8	11	8.3	17	8.5	
65–74	8	11.8	8	6.0	16	8.0	
≥ 75	6	8.8	6	4.5	12	6.0	
**Co-detection**							
No	57	83.8	109	82.0	166	82.6	0.893
Yes	11	16.2	24	18.0	35	17.4	
**Admission**							
No admission	36	52.9	52	39.1	88	43.8	0.085
Admission	32	47.1	81	60.9	113	56.2	
**Hospital admission time (days)**							
Median (IQR)	4.0 (2.0–5.2)		4.0 (2.0–6.0)		4.0 (2.0–6.0)		0.504
**ICU**							
No	30	93.8	72	88.9	102	90.3	0.665
Yes	2	6.2	9	11.1	11	9.7	

ICU: intensive care unit; IQR: interquartile range.

All diagnostic tests were performed by the Regional Public Health Laboratory Kennemerland (RPHLK). The p values for continuous variables were calculated with the Mann-Whitney U-test, for categorical variables with a Pearson’s chi-squared test. We defined significance at p < 0.05.

Of the 133 patients, 81 (60.9%) were admitted to the hospital, including 30 children (37.0%); for the detailed data by age group and admission we refer to Supplementary Table S2. Three patients had extrapulmonary manifestations, all other patients were admitted with pneumonia/pulmonary infection. The median duration of admission was 4 days (IQR: 2–6 days). Children had a significantly shorter median duration of admission than adults (2.5 vs 5 days; p < 0.001). Nine adult patients (11.1%) were admitted to the ICU, of whom three needed mechanical ventilation. The reason for ICU admission was hypoxia in seven of them. Four patients had underlying comorbidities that may have contributed to the ICU admission (i.e. lung cancer, cardiovascular disease, diabetes, alcohol and drug use disorder, and neurological disorder). Twenty-four (18.0%) patients with *M. pneumoniae* had a co-detection; the majority with rhinovirus (n = 10) and respiratory syncytial virus (RSV) (n = 6); no significant difference was found between children and adults.

In comparison, we found a total of 68 *M. pneumoniae* detections in 2019 and 2020. During those years, the median age was significantly higher: 40 years (IQR: 21–57) compared with 28 years in 2023 (IQR: 9–43; p = 0.005). In 2023, we observed a higher percentage of admissions, although the difference was not statistically significant; the percentages of ICU admissions and the duration of admission were not significantly different either (Table).

## Incidence of *Mycoplasma pneumoniae* in the Netherlands

**Figure 2:**
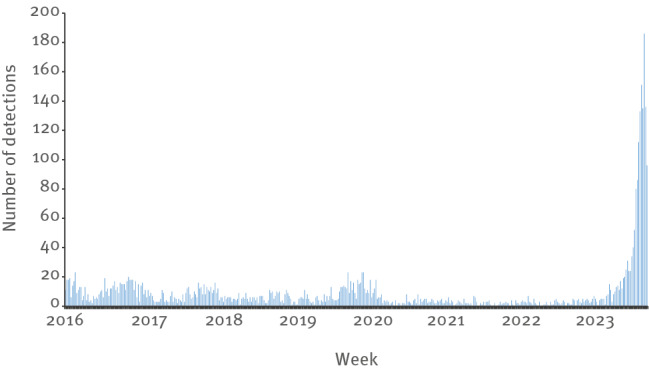
*Mycoplasma pneumoniae* detections, the Netherlands, 2016–2023 (n = 3,857)

Source: virologische weekstaten, a surveillance system from the Dutch National Institute for Public Health and the Environment.

To analyse the national trend of *M. pneumoniae* infections, we used data from the ‘*virologische weekstaten’*, a surveillance system from the Dutch National Institute for Public Health and the Environment (RIVM) which receives data from the Dutch Working Group on Clinical Virology from the Dutch Society for Clinical Microbiology [6]. The RIVM receives data on the number and type of pathogen detections without any demographic data of the patients or information on the detection method. In 2023, a total of 1,586 *M. pneumoniae* detections were observed in *virologische weekstaten;* in week 50 of 2023, there were 186 detections, compared with 40 detections in week 42, a more than 4-fold rise in new detections (Figure 2). In contrast, during 2019 and 2020 a combined total of 688 *M. pneumoniae* were detected; the data until 2022 can be accessed in higher resolution in Supplementary Figure S2.

In addition, we described data from ‘*Infectieradar’*, a digital participatory syndromic surveillance system by the RIVM that has been collecting data on respiratory symptoms from the general population since 2020; it includes participants older than 16 years and resident in the Netherlands who report their symptoms weekly [7]. Since October 2022, a random subset of 200 participants experiencing acute respiratory symptoms have been instructed to self-sample with a NP/OP swab. These swabs are analysed weekly for respiratory pathogens [8] at the RIVM. In the *Infectieradar,* a total of 6,387 self-tests were analysed in 2023. Starting from July 2023, *M. pneumoniae* was detected in 41 participants, of which 31 were detected between 1 November and 31 December (weeks 44–52). Between October 2022 and June 2023, *M. pneumoniae* was not detected in any of the participants.

## Discussion

Starting in late summer 2023 (week 43), we observed a steep rise in *M. pneumoniae* detections in Spaarne Gasthuis hospital. This is consistent with the overall increase in the incidence of *M. pneumoniae* detections in the Netherlands, with a 4-fold rise in week 50 compared with week 42. At the time of writing, the number of detections in the Netherlands is much higher than in previous years; in 2019 and 2020, there was a period of elevated incidence in Spaarne Gasthuis hospital, but the national detection rate of *M. pneumoniae* was within the range of the previous 4 years [9]. The 2023 detection rate in the Netherlands has risen beyond the total number of the epidemic in 2011 which had 916 detections with a peak incidence in week 50 [10]. We observed a high number of hospital admissions due to *M. pneumoniae* infection. However, the proportion of hospital admissions is not statistically significant different from 2019 and 2020.

The ESGMAC MAPS study, a global surveillance study of *M. pneumoniae*, showed an increasing incidence in the period from 1 April to 30 September 2023 with a mean incidence of 4.12% in April compared with 0.82% in March 2023 [11]. Most detections were from Denmark. Denmark's Statens Serum Institute (SSI) recently published that their infection numbers had risen to epidemic levels in week 43 (October) 2023 [12]. These data are consistent with the situation in our region and in the Netherlands.

The reason for this increase in incidence is not yet known but it is probably related to both pathogen- and host-related factors. The cycle of *M. pneumoniae* occurrence is speculated to be caused by shifts in the dominant strain [13]. The current upsurge might fit the normal cycle, with a long periodicity for the dominant strain to shift. However, it may also be related to host factors resulting from the non-pharmaceutical interventions such as stay-at-home orders during the COVID-19 pandemic. The interventions caused a decline in circulating respiratory pathogens (including *M. pneumoniae*) [14]. A resulting trend of waning herd immunity, also known as ‘immunity debt’, has been described [15], resulting in a population more susceptible to respiratory pathogens. Another possible explanation might be that since the COVID-19 pandemic, there is a tendency to more frequent testing of patients with respiratory symptoms [16], resulting in more pathogen detections. However, at Spaarne Gasthuis, 3,227 tests were performed in 2023, which does not differ from the combined 6,889 tests in 2019 and 2020.

At Spaarne Gasthuis hospital, the patients with *M. pneumoniae* in November and December 2023 had a lower median age than those in 2019 and 2020 (28 vs 40 years). This is in line with a global increase in respiratory tract infections in children [17,18]. Part, but not all, of this increase seems to be caused by *M. pneumonia*e infections. Another part is due to circulating known pathogens such as RSV, influenza virus and severe acute respiratory syndrome coronavirus 2 (SARS-CoV-2). 

Current Dutch guidelines recommend treatment of *M. pneumoniae* infections with macrolide or tetracycline antibiotics for adults [19]. Even though there is an increase in *M. pneumoniae* infections, we would like to caution against empiric treatment of community-acquired pneumonia with macrolides. Macrolide resistance in *M. pneumoniae* is relatively low in many countries in Europe [20]. To prevent the resistance rates from rising, restricted use of macrolides is import, also in light of the rising macrolide resistance of *Streptococcus pneumonia* [21]. Moreover, for school-aged children, it is unclear whether macrolides have a beneficial effect [22] Since *M. pneumoniae* infections are generally mild, the advice for empirical treatment of pneumonia remains to start with beta-lactam antibiotics. If there is no response to first-line treatment or a positive PCR for *M. pneumoniae,* the advice is to switch to macrolides or tetracycline [19].

There are several limitations regarding the interpretation of our data. Firstly, these are observational data which may be influenced by selection bias (e.g. different test strategies in different age categories). Secondly, detection of *M. pneumoniae* by PCR in NP/OP swabs is not synonymous to infection; high carriage rates have been described in some studies in children, but so far not in studies with (young) adults [23-25]. Thirdly, we mainly analysed data of people with respiratory symptoms. It is important to emphasise that although the major clinical presentation is pulmonary infection, presentations with extrapulmonary manifestations can occur, such as erythema exsudativum multiforme and encephalitis [1]. Fourthly, the national data did not include any demographic data on the patient or the severity of symptoms. Moreover, since the number of tested samples of the *virologische weekstaten* is unknown, more testing could have led to more positive samples. Finally, for in-depth analysis of the current elevated levels of *M. pneumoniae* detection, we only had detailed data from a small region of the Netherlands which may not be representative of the rest of the country. However, we are confident that all data from various sources taken together represent a realistic overview of the current upsurge of *M. pneumoniae* detections.

## Conclusion

Currently, we observe a local and national *M. pneumoniae* upsurge in the Netherlands. The incidence is higher than reported in previous years and has not been that high since 2011. The median age of the patients in 2023 was lower than in 2019 and 2020 (28 vs 40 years). *M. pneumoniae *should be considered in patients with respiratory symptoms, especially children. To understand the current increased incidence in more detail, data on e.g. severity of disease, circulating strains and degree of macrolide resistance are important. 

## Supplementary Data

763000 Supplement
