# Supplementary material for: Increased incidence of Mycoplasma pneumoniae infections and hospital admissions in the Netherlands, November to December 2023
Source: Euro Surveill. 2024 Jan 25;29(4):2300724. doi: 10.2807/1560-7917.ES.2024.29.4.2300724 (PMC10986650; doi:10.2807/1560-7917.ES.2024.29.4.2300724)
Supplement: Supplement [file 23-00724_LELYVELD_Supplement.pdf]

Supplementary materials

Increased incidence of *Mycoplasma pneumoniae* infections and hospital admissions in the Netherlands, November–December 2023

Disclaimer: *This supplementary material is hosted by Eurosurveillance as supporting information alongside the article "Increased incidence of Mycoplasma pneumoniae infections and hospital admissions in the Netherlands, November–December 2023", on behalf of the authors, who remain responsible for the accuracy and appropriateness of the content. The same standards for ethics, copyright, attributions and permissions as for the article apply. Supplements are not edited by Eurosurveillance and the journal is not responsible for the maintenance of any links or email addresses provided therein.*

Table S1. Patient demographics of Mycoplasma pneumoniae detections in the Spaarne Gasthuis hospital Hoofddorp/Haarlem, the Netherlands. Data of 2023 (n=133), compared to the last episode of increased incidence in 2019/2030 (n=68), difference shown between age above and below 18 years. All diagnostic test were performed by the Regional Public Health Laboratory Kennemerland (RPHLK).

		age < 18	age >= 18	Total	p
Gender	Male	27 (49.1)	38 (48.7)	65 (48.9)	1.000
	Female	28 (50.9)	40 (51.3)	68 (51.1)	
Admission time	Median (IQR)	2.5 (2.0 to 4.0)	5.0 (3.0 to 7.0)	4.0 (2.0 to 6.0)	<0.001
IC	No	30 (100.0)	42 (82.4)	72 (88.9)	0.038
	Yes	0 (0.0)	9 (17.6)	9 (11.1)	
Co-infection	No	42 (76.4)	67 (85.9)	109 (82.0)	0.238
	Yes	13 (23.6)	11 (14.1)	24 (18.0)	
Co-infection with rhino and rsv	No or other co-detection	47 (85.5)	70 (89.7)	117 (88.0)	0.633
	Rhino and RSV co-detection	8 (14.5)	8 (10.3)	16 (12.0)	
Admission	No admission	25 (45.5)	27 (34.6)	52 (39.1)	0.280
	Admission	30 (54.5)	51 (65.4)	81 (60.9)	

p-values for contitous variables were calculated with a test, for categorigal variables with a Pearson's Chi-squared test. We defined significance at a p-value <0.05

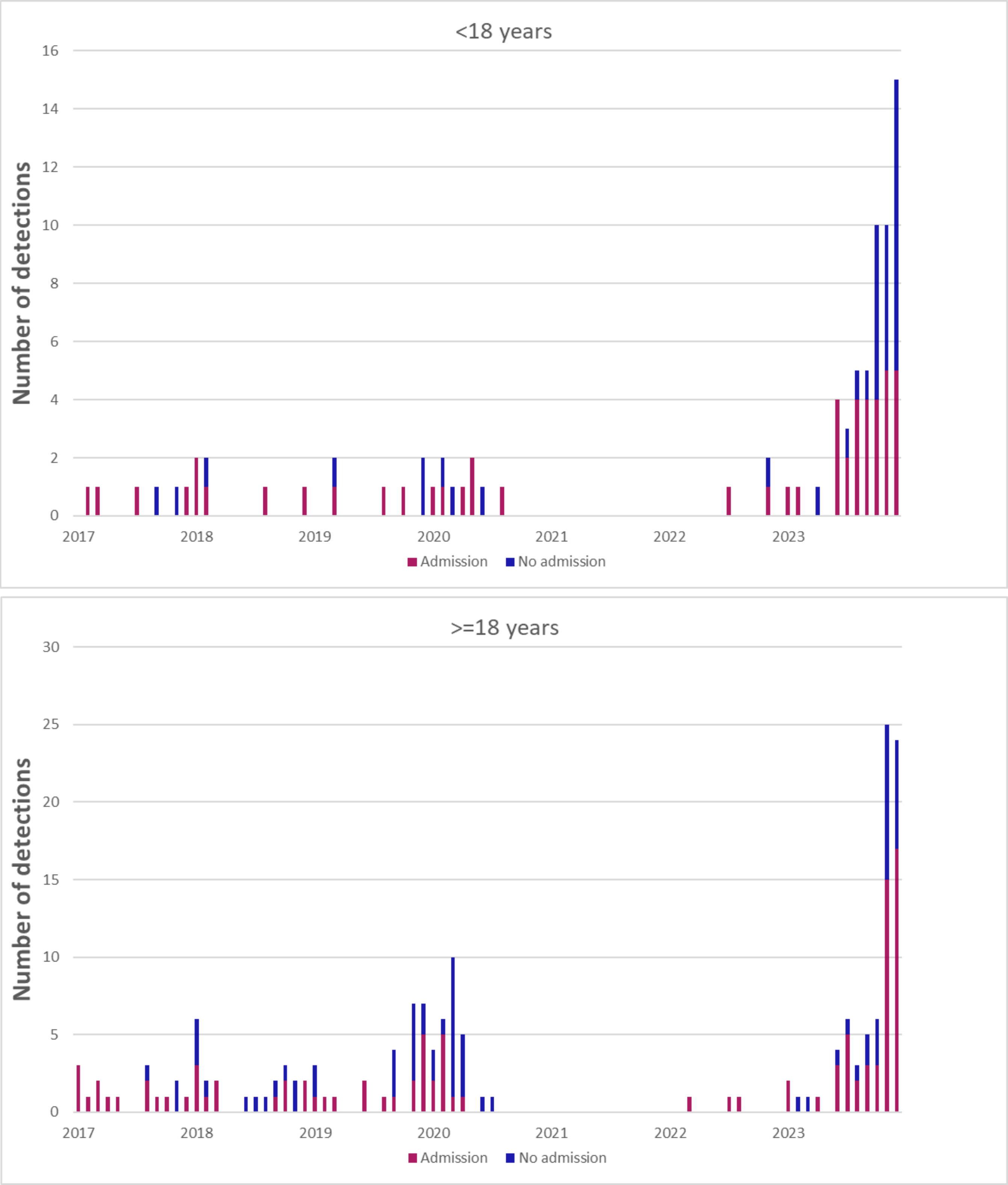

Figure S1. Mycoplasma pneumoniae detections in the Spaarne Gasthuis hospital, Hoofddorp/Haarlem, the Netherlands, 2017-2023. Difference shown between age above and below 18 years. *M. pneumoniae* detections were done by PCR on naso- and/or oropharyngeal swab (Fourie et al. 2023).

Table S2. Patient demographics of Mycoplasma pneumoniae detections in the Spaarne Gasthuis hospital Hoofddorp/Haarlem, the Netherlands. Data of 2023 (n=133), compared to the last episode of increased incidence in 2019/2030 (n=68), difference shown between admission and no admission. All diagnostic test were performed by the Regional Public Health Laboratory Kennemerland (RPHLK).

		**2019-2020**			
		No admission	Admission	Total	p-value
Gender	Male	13 (36.1)	19 (59.4)	32 (47.1)	0.094
	Female	23 (63.9)	13 (40.6)	36 (52.9)	
Age	Median (IQR)	41.5 (25.8 to 62.2)	36.0 (10.8 to 48.2)	40.0 (21.2 to 57.0)	0.302
Age group	0-4	3 (8.3)	2 (6.2)	5 (7.4)	0.305
	5-11	1 (2.8)	7 (21.9)	8 (11.8)	
	12-17	2 (5.6)	0 (0.0)	2 (2.9)	
	18-29	4 (11.1)	3 (9.4)	7 (10.3)	
	30-39	5 (13.9)	6 (18.8)	11 (16.2)	
	40-49	8 (22.2)	7 (21.9)	15 (22.1)	
	50-64	4 (11.1)	2 (6.2)	6 (8.8)	
	65-74	6 (16.7)	2 (6.2)	8 (11.8)	
	> 75	3 (8.3)	3 (9.4)	6 (8.8)	
Admission time	Median (IQR)		4.0 (2.0 to 5.2)	4.0 (2.0 to 5.2)	-
IC	No		30 (93.8)	30 (93.8)	-
	Yes		2 (6.2)	2 (6.2)	
Co-infection	No	27 (75.0)	30 (93.8)	57 (83.8)	0.077
	Yes	9 (25.0)	2 (6.2)	11 (16.2)	
		**2023**			
		No admission	Admission	Total	p-value
Gender	Male	16 (30.8)	49 (60.5)	65 (48.9)	0.002
	Female	36 (69.2)	32 (39.5)	68 (51.1)	
Age	Median (IQR)	20.0 (8.8 to 38.2)	30.0 (10.0 to 50.0)	28.0 (9.0 to 43.0)	0.081
Age group	0-4	8 (15.4)	6 (7.4)	14 (10.5)	0.306
	5-11	11 (21.2)	16 (19.8)	27 (20.3)	
	12-17	6 (11.5)	8 (9.9)	14 (10.5)	
	18-29	6 (11.5)	10 (12.3)	16 (12.0)	
	30-39	9 (17.3)	13 (16.0)	22 (16.5)	
	40-49	8 (15.4)	7 (8.6)	15 (11.3)	
	50-64	1 (1.9)	10 (12.3)	11 (8.3)	
	65-74	2 (3.8)	6 (7.4)	8 (6.0)	
	> 75	1 (1.9)	5 (6.2)	6 (4.5)	
Admission time	Median (IQR)		4.0 (2.0 to 6.0)	4.0 (2.0 to 6.0)	-
IC	No		72 (88.9)	72 (88.9)	-
	Yes		9 (11.1)	9 (11.1)	
Co-infection	No	37 (71.2)	72 (88.9)	109 (82.0)	0.018
	Yes	15 (28.8)	9 (11.1)	24 (18.0)	

p-values for contitous variables were calculated with a test, for categorigal variables with a Pearson's Chi-squared test. We defined significance at a p-value <0.05

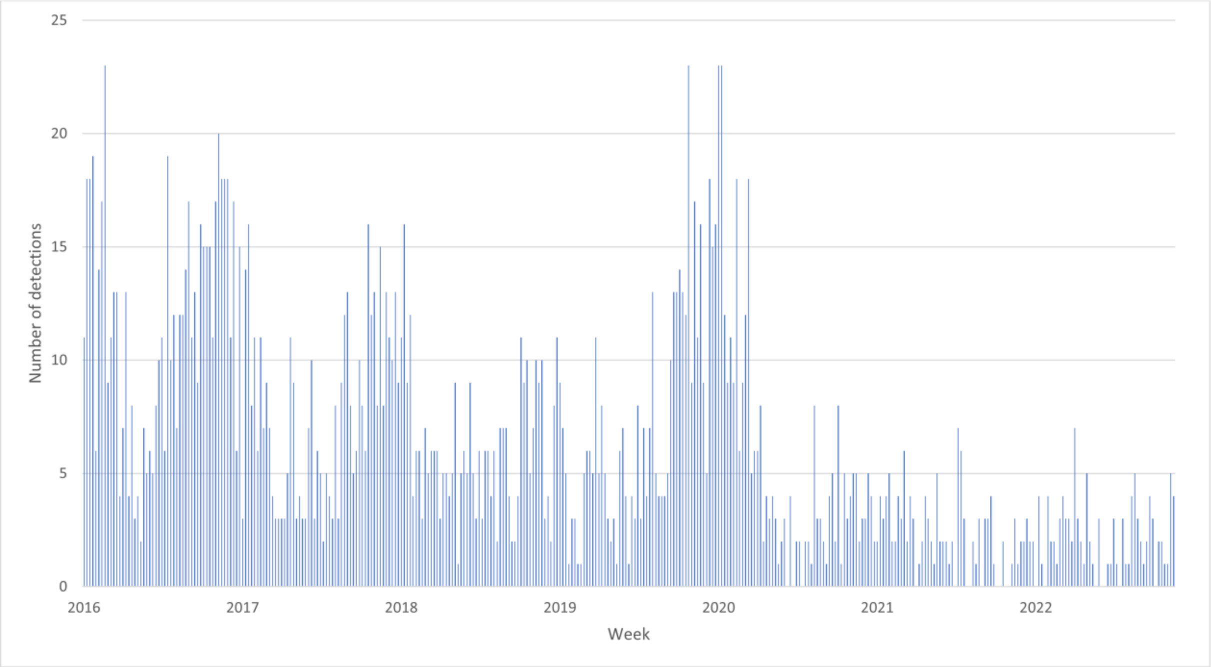

Figure S2. Mycoplasma pneumoniae detections in the "*virologische weekstaten";* a surveillance system from the Dutch National Institute for Public Health and the Environment (RIVM), the Netherlands, 2016-2022
